# Optimal location of water level sensors for monitoring mine water inrush based on the set covering model

**DOI:** 10.1038/s41598-021-82121-7

**Published:** 2021-01-29

**Authors:** Qiang Wu, Zhili Du, Yingwang Zhao, Hua Xu, Xiaoyan Zhang

**Affiliations:** 1 College of Geoscience and Surveying Engineering, China University of Mining and Technology (Beijing), Beijing, China; 2 National Engineering Research Center of Coal Mine Water Hazard Controlling, Beijing, China; 3 Information Engineering College, Beijing Institute of Petrochemical Technology, Beijing, China

**Keywords:** Hydrology, Energy science and technology, Engineering

## Abstract

Water inrush is one of the major mining disasters that may lead to numerous casualties. The development of information techniques makes it possible to monitor the occurrence and evolution of water inrush. Then, locating monitors for water inrush becomes a primary problem. This study presents a method of optimal location of water level sensors by constructing a set covering model. The monitoring scope of the water level sensor at each location in a given time is computed first based on the numerical simulation of water spreading along mine tunnels. In this simulation, the water inrush quantity is assigned using the mine drainage capability over which an accident may occur. Then the greedy algorithm is used to optimize the number and positions of water level sensors. As results, a mine water disaster can be monitored in the given time after it happened. The proposed method is then verified in the Beiyangzhuang coal mine in the North China. The results show that at least 22, 36, 42, 64 and 106 water level sensors are needed to monitor water disasters in the whole mine within 60, 30, 20, 10 and 5 min, respectively.

## Introduction

Coal is the main energy resource in China, and coal mines exist in many parts of the country. Hydrogeological conditions in some coal mines are extremely complex, and these coal mines are frequently threatened by a variety of hydrogeological hazards. Among all the hazards in coal mines, water inrush is one of the most notorious^1–5^. Between 2007 and 2017, the number of accidents and casualties caused by mine water inrush declined in general. However, the average number of deaths per accident did not decrease significantly, and even increased in 2014 and 2015. The main reason for this situation is that it is unable to accurately predict the time and locations of mine water inrush disasters. Once a water disaster occurs, effective emergency rescue measures cannot be taken in time due to the lack of accurate first-hand information of the water disaster.

In recent years, the monitor of water inrush becomes a hotspot. The nation has begun to put forward the development concept of "people-oriented", and has paid more and more attention to people. The latest” Detailed Rules for Coal Mine Water Prevention and Control” propose to firmly establish the concept of people-oriented and safe development, and point out in Chapter 7 that emergency response to water disasters should be targeted, scientific and operable^7^. Its essence is to monitor the occurrence of mine water disaster as soon as possible and provide accurate and reliable information in time for emergency rescue. Fortunately, it becomes possible to realize the real-time monitoring of water disaster by setting water level sensors in mine tunnels with the continuous development of social informatization, the progress of monitoring technology, the reduction of monitoring equipment costs, and the improvement of monitoring network technology, such as 5G network technology. Then, it becomes an urgent engineering problem to arrange water level sensors in the mine tunnels to monitor the occurrence of water disaster quickly and obtain accurate first-hand information in time, such as the quantity, location and spread area of the water disaster. At present, the monitoring system mainly monitors the amount of water passing through the mine drainage pipelines. The monitor sensors are located based on single factor or static monitoring, and even only monitoring the suspicious areas of coal seam floor at regular and fixed points in a few water hazards monitoring^8,9^. There is still little research on real-time monitoring of water flow in the mine tunnels, especially for the monitoring of water flow spreading in the tunnels after water inrush.

As a valuable method for siting service facilities, facility location models have been widely used in numerous applications, including determining the location of defense facilities considering a time horizon^10^, optimal placement of sensors in watercourses^11^, determining the location and size of charging stations for electric vehicles^12^, positioning distribution centers for emergency stockpiles to improve preparedness in the event of a disaster^13^, positioning water quality monitoring stations^14^, solving hub location problems in urban transport and liner shipping network design^15^, designing supply chain networks^16^, choosing the location of facilities in a humanitarian relief chain responding to quick-onset disasters^17^, and examining service systems applied to the distribution of policemen in highway networks^18^. Covering problems are among the most popular facility location models. They have always been very attractive for research due to their real-world applicability, especially for emergency facilities^19–33^.

In this paper, a method to optimally locate of water level sensors for mine water inrush is presented based on the set covering model using the simulation data of mine water inrush process. These sensors can record the depth of water in tunnels. Furthermore, the monitoring data of water level sensors can be analyzed for further prediction and prevention of mine water inrush so as to carry out effective emergency rescue, and reduce casualties and property losses.

The main highlights of this paper are as follows:

1. This study innovatively abstracts the optimal location of water level sensors into the set covering model. The monitoring scope of the water level sensor in each position is different along the tunnels, and it is necessary for optimally locating water level sensors.
2. In this paper, the capacity of drainage system is taken as the quantity of simulated water inrush for the first time, so as to obtain the monitoring scope of the water level sensors.
3. Greedy algorithm is used to solve the set covering model. And it achieves the real-time monitoring of water disasters in the whole mine with the least sensors in the given time.

## Establishment of the set covering model

As can be seen from the previous introduction, the set covering model is one of the most popular facility location models. It is widely used in the location of various facilities in the real world, especially for emergency facilities^20–31^. In the optimally locating problem of water level sensors for monitoring mine water inrush, the goal is to monitor water inrush in the whole mine with the least sensors within *T* minutes. Parameter *T* is the monitoring response time, that is, once a water inrush occurs in the mine, it will be monitored within *T* minutes. The monitoring scope of a water level sensor refers to a certain area where the occurred water inrush can be monitored by the sensor within *T* minutes. If the whole mine is represented as a set *N* and the monitoring scope of a water level sensor is represented as a set *m*, the monitoring scopes of all the water level sensors in each position can be represented as *M*. Therefore, the optimal location of the water level sensors can be abstracted as the set covering model:

Select the least *m* from the *M* to completely “cover” the set *N*.

The monitoring scope of water level sensors in each position will be different along the tunnels, and it is necessary for optimally locating water level sensors. The following will explain in detail how to construct the mine tunnels’ topology (the set *N*) and obtain the monitoring scopes (the *M*).

### Construction of the mine tunnels’ topology

The mine tunnels’ topology is established on the basis of the distribution of mine excavation engineering and mine tunnels’ traverse points. The traverse points are abstracted into nodes with coordinates (*x*, *y*, *z*), and the water flow at these nodes is recorded during simulation of the water inrush process, including the time when water arrives and the depth of the water at different times. In the mine tunnels’ topology, the nodes are numbered uniformly, starting from 0. Assuming that the number of nodes in the mine tunnel topology is *n*, the set of all nodes can be expressed as $N=\{\mathrm{0,1},2,\dots , n-1\}$. The number of nodes can be increased by interpolation to record the water flow dynamics in more detail.

**Figure 1:**
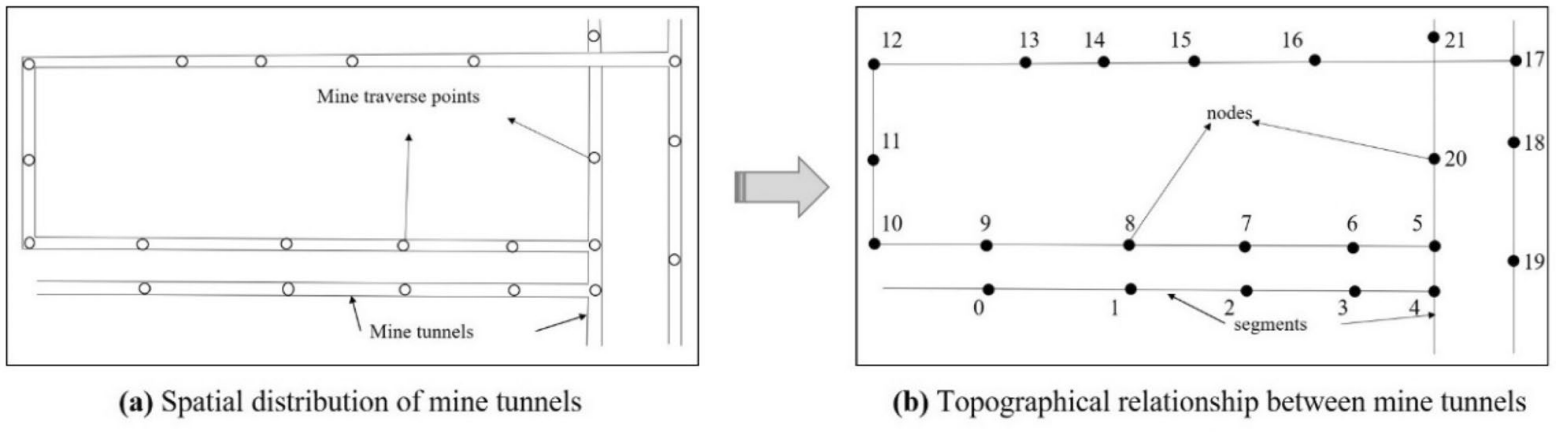
Construction of topological structure of coal mine tunnels.

The tunnel between two nodes is abstracted into a line segment. The attributes of each line segment include the cross-sectional area, length, slope of the tunnel and the connections between tunnels. Figure 1 shows the process to establish the topological structure of a coal mining face and an excavation tunnel in a mine.

### The spreading scope of water inrush

Water inrush process can be simulated using the Storm Water Management Model (SWMM) software based on the topology of the mine tunnels. It is an opensource module that has been widely used in the simulation of groundwater flow^34,35^. Water inrush process can be simulated as one-dimensional, unsteady and non-uniform open-channel flow^38^. A large number of simulation data of water inrush process at the nodes of the mine tunnels’ topology can be obtained by SWMM software.

The inrush quantity is of importance for numerical simulation as the boundary condition. In this paper, the quantity of water inrush is set according to the drainage capacity. There is a complete drainage system in the coal mine, and the drainage capacity of each mining area or mining face is different. For example, when the water inrush process is simulated at node *i*, if the drainage capacity of the area is *Q*, and thus the quantity of the simulated water inrush is set to *Q*. It is because when the quantity of the water inrush is less than *Q*, it can be pumped out in time without causing water inrush hazards. It is of practical significance to timely monitor the occurrence of water inrush with a water inrush quantity greater than or equal to *Q*. Furthermore, if a water inrush accident can be monitored whose quantity is *Q*, then it can monitor whose quantity is greater than *Q* in less time.

**Figure 2:**
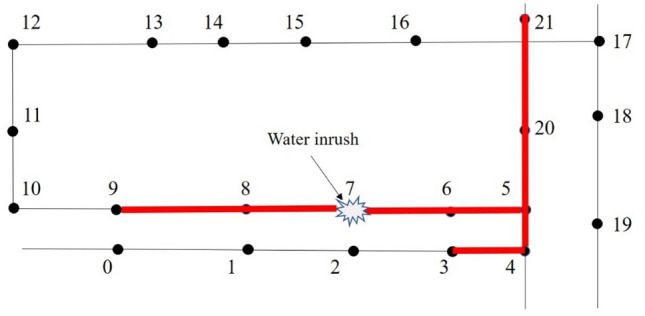
The spreading scope of water inrush at node 7.

The spreading scope with *T* minutes after a water inrush happened can be obtained by numerical simulation. Figure 2 shows a water inrush occurred at node 7. The water flow extends to nodes 3, 9, and 21 after 60 min. The spreading scope of node 7 is the area marked in the figure by red lines.

### Determination of monitoring scope

Water inrush occurring at node 7 can be monitored within 60 min if a water level sensor is located anywhere within the red area in Fig. 2. Hence, if a water level sensor is placed within the spreading scope of node *i*, the water inrush happened at node *i* can be monitored within *T* minutes.

**Figure 3:**
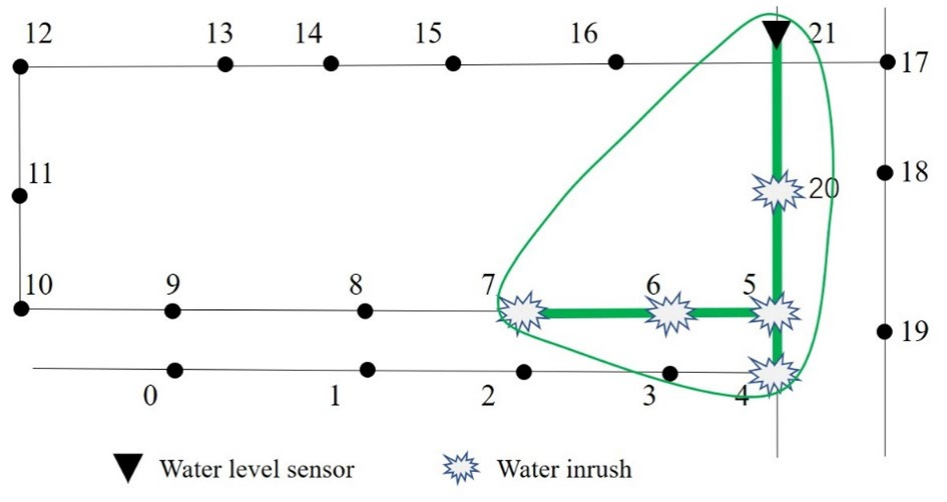
The monitoring scope of the water level sensor at node 21.

Assuming that the spreading scope of node *m* coverages node *j*, a water level sensor placed at node *j* can monitor the water inrush happened at node *m* and the segments between them. As shown in Fig. 3, node 21 exists within the spreading scopes of nodes 4, 5, 6, 7, 20 and 21. The monitoring scope of the water level sensor placed at node 21 is the area marked by the green line. The monitoring scope of the water level sensor placed at node *j* can be expressed as *M*_*j*_. The value of *M*_*j*_ is obtained by counting the number of spreading scopes where node *j* locates. The schematic diagram of *M*_21_ is shown in Fig. 3. It can also be expressed in the form of a set: ${M}_{21}=\left\{\mathrm{4,5},\mathrm{6,7},\mathrm{20,21}\right\}$.

The monitoring scope of water level sensors placed at each node can be obtained through the spreading scope of water inrush of all nodes. The monitoring scope of all nodes can be represented as *M*.

$$M=\left\{{M}_{0}, {M}_{1}, {M}_{2}, \dots ,{M}_{j}\right\} j=(0, 1, 2, \dots , n-1)$$

The problem of optimally locating water level sensors then has been transformed into the set cover problem, which can be expressed as selecting the smallest number of monitoring scopes *M*_*j*_ from *M* to “cover” the set *N*. The detailed steps to solve this problem are explained in the next section.

## Solution procedures

**Figure 4:**
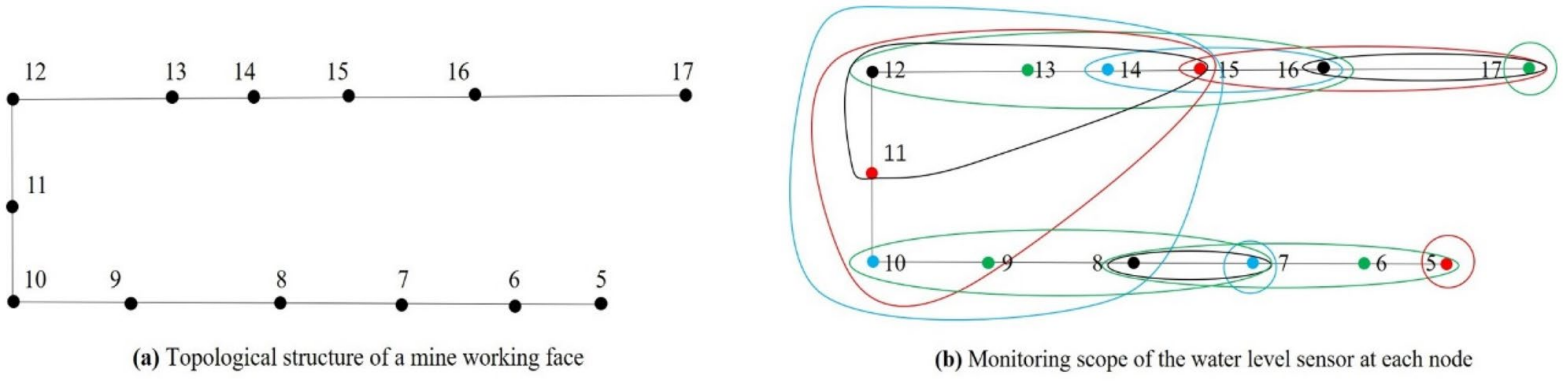
Monitoring scope of each node in a mine working face.

In this section, the optimal location method of water level sensors is realized based on the above set covering model using the greedy algorithm. Greedy algorithm refers to the best or optimal (that is, the most advantageous) choice is taken in each step of the selection when solving the problem, so as to hope that the result is the best or optimal algorithm. The results obtained by the greedy algorithm are often not the optimal results (sometimes the optimal solution), but they are relatively approximate (close to) the optimal solution. The advantages of greedy algorithm in solving the set covering model are simple, fast and easy to operate^37–40^. A model of a single coal mining face is established to illustrate the detailed steps. The set of all nodes is expressed as $N=\{5, 6, 7, \dots , 17\}$, as shown in Fig. 4a. The capacity of the drainage system in the mining face is 800 m^3^/h. The process of water inrush at each node is then simulated with the quantity, 800 m^3^/h. The time of the water inrush processes is set to 60 min. The monitoring scopes of all nodes are represented as *M*.

$$M=\left\{{M}_{5}, {M}_{6},{M}_{7}, \dots ,{M}_{j}\right\} j=(5, 6, 7, \dots , 17)$$

By analyzing and processing the spreading scopes of the 13 nodes, *M* can be obtained. Then, the *M* are represented with different colors in Fig. 4b. Each node and its corresponding monitoring scope use the same color.

**Figure 5:**
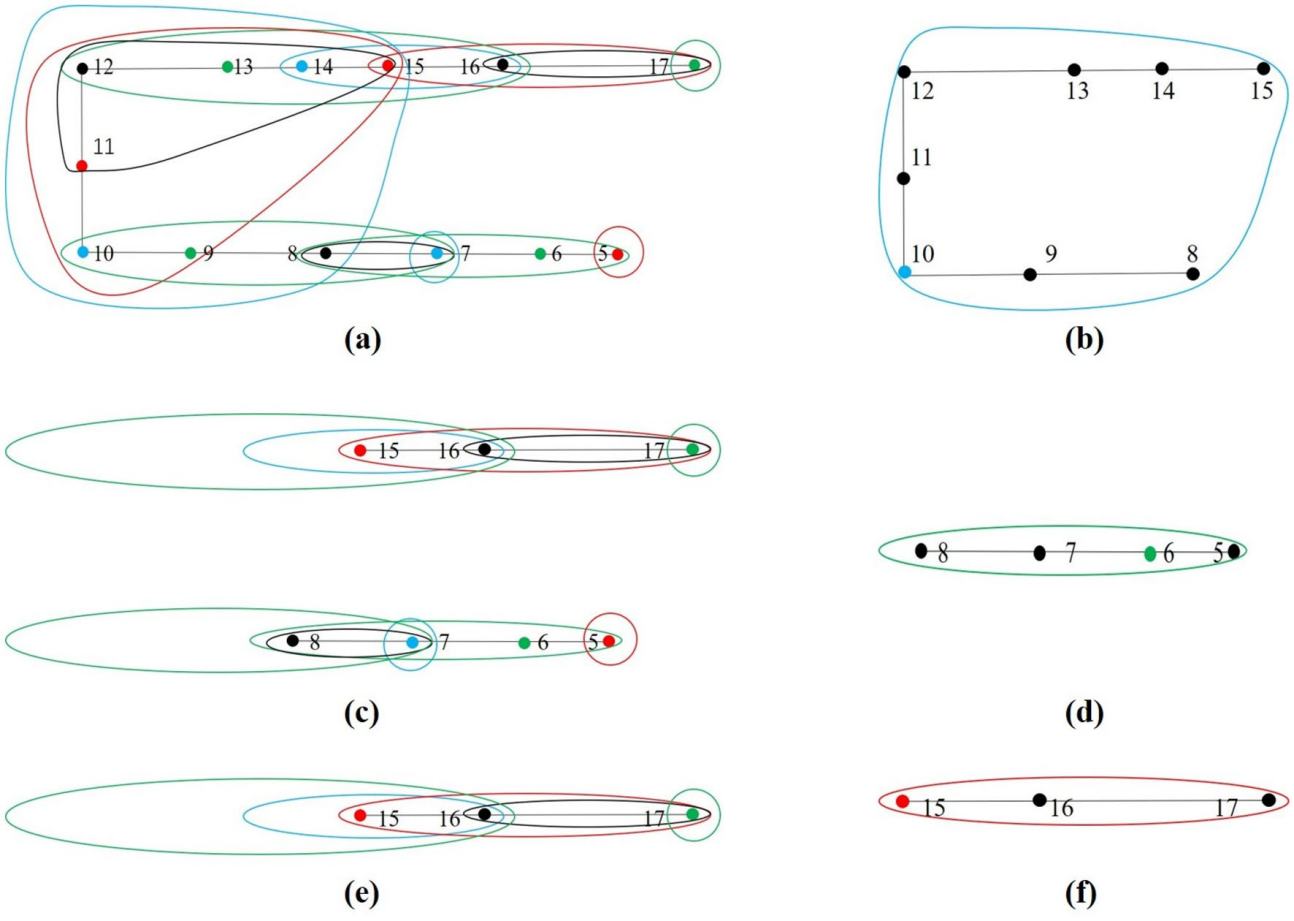
The specific steps of the greedy algorithm.

Then the set covering model of this mining face be expressed as selecting the smallest number of monitoring scopes *M*_*j*_ from the *M* to “cover” the set *N*. The specific steps required to solve this model by the greedy algorithm, as illustrated in Fig. 5, are as follows.

1. Calculate the number of nodes in each monitoring scope and select the biggest one with most nodes. As shown in Fig. 5b, ${M}_{10}=\left\{\mathrm{8,9},\mathrm{10,11,12,13,14,15}\right\}$ contains most nodes and becomes the first selected one.
2. Delete the nodes contained in *M*_10_ from the set *N*, then $N=\{5,\mathrm{ 6,7},\mathrm{16,17}\}$, and delete any monitoring scopes that does not contain the nodes in the set *N*. The result of this step is shown in Fig. 5c.
3. Repeat step 1 to obtain the location of the next water level sensor at node 6. The *M*_6_ is shown in Fig. 5d, ${M}_{6}=\left\{\mathrm{5,6},\mathrm{7,8}\right\}$.
4. Repeat step 2 to obtain the result shown in Fig. 5e, $N=\{\mathrm{16,17}\}$.
5. Repeat steps 1 and 2 to obtain the location of the third water level sensor at node 15. The *M*_15_ is shown in Fig. 5f, ${M}_{15}=\left\{\mathrm{15,16,17}\right\}$.As results, the set *N* is empty, and the set *N* has been covered, then the process of optimizing the location of sensors is completed.

**Figure 6:**
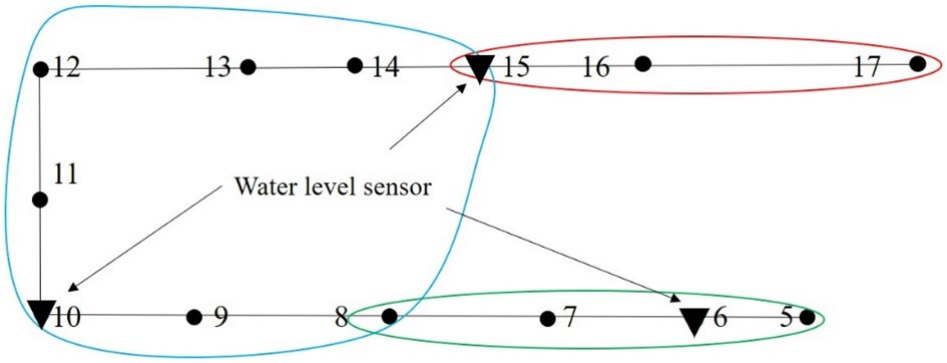
The result of the greedy algorithm (node 10, 6, and 15).

*M*_10_, *M*_6_, and *M*_15_ are finally selected using this optimal location method, as shown in Fig. 6. Water level sensors are therefore located at nodes 10, 6, and 15, which can realize timely monitoring of water inrush occurring in this working face within 60 min. This optimal location method can be still applicable when the research area is extended to the whole mine.

## Case study

### Study area

**Figure 7:**
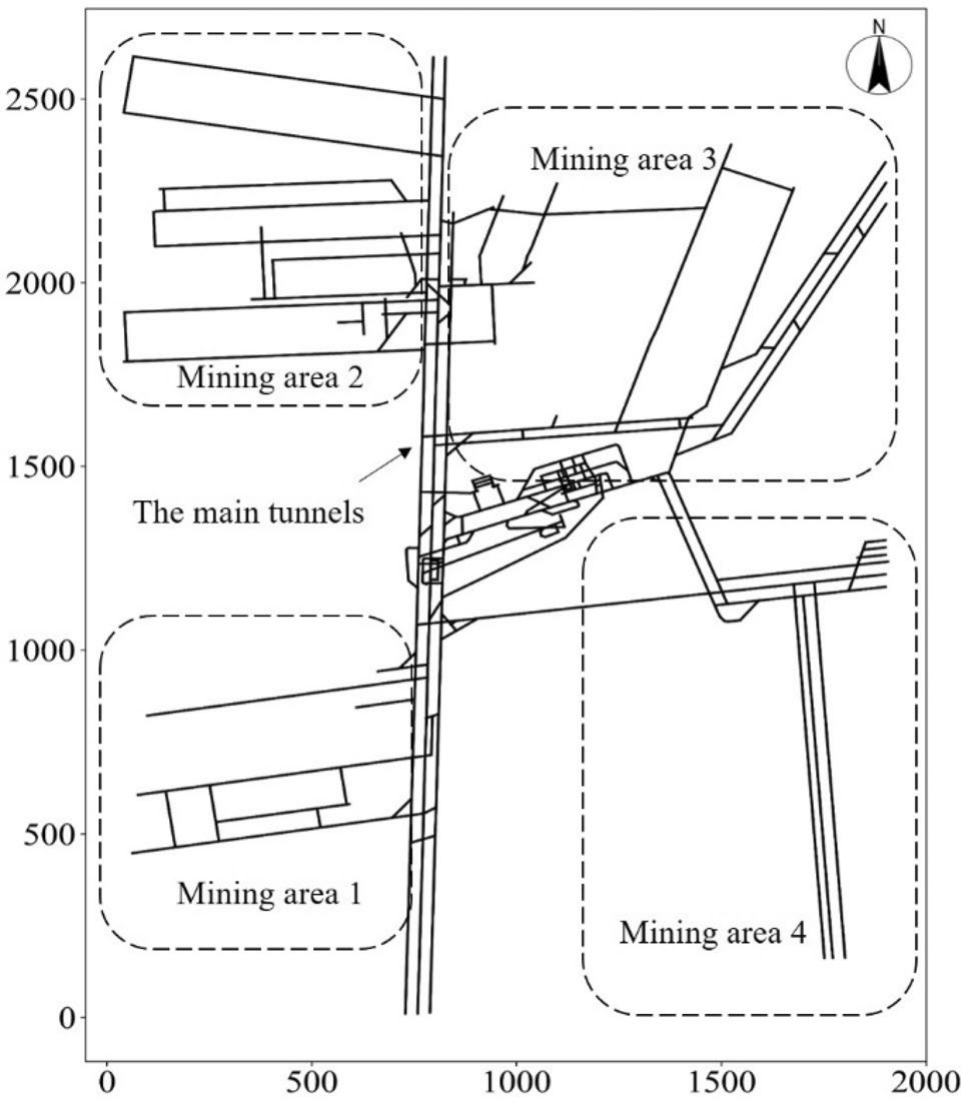
Distribution of mining areas in Beiyangzhuang mine.

Beiyangzhuang Mine Field is located in the southeast of Yuxian Mining Area, Hebei Province. It is about 10 km long north to south, 5–8 km wide east to west, and has an area of 52 km^2^ (see Fig. 7). For the sake of confidentiality, relative coordinates were used in this paper. The main coal seam in the mine is the No. 5 Coal Seam of the Lower Jurassic Xiahuayuan Formation, which is affected by the Ordovician karst water. The hydrogeological conditions in Beiyangzhuang coalmine are complex.

There is a central drainage pumping station and a freshwater pumping station at the bottom of the auxiliary shaft in Beiyangzhuang mine. The drainage capacity of the mine is 5080 m^3^/h. The mine is divided into four mining areas (Fig. 7). The drainage capacities of mining areas 1, 2, 3, and 4 are 800 m^3^/h, 1000 m^3^/h, 1200 m^3^/h, and 2000 m^3^/h, respectively. The remaining areas, including the main tunnels, have a drainage capacity of 5080 m^3^/h.

### Optimal location method

**Figure 8:**
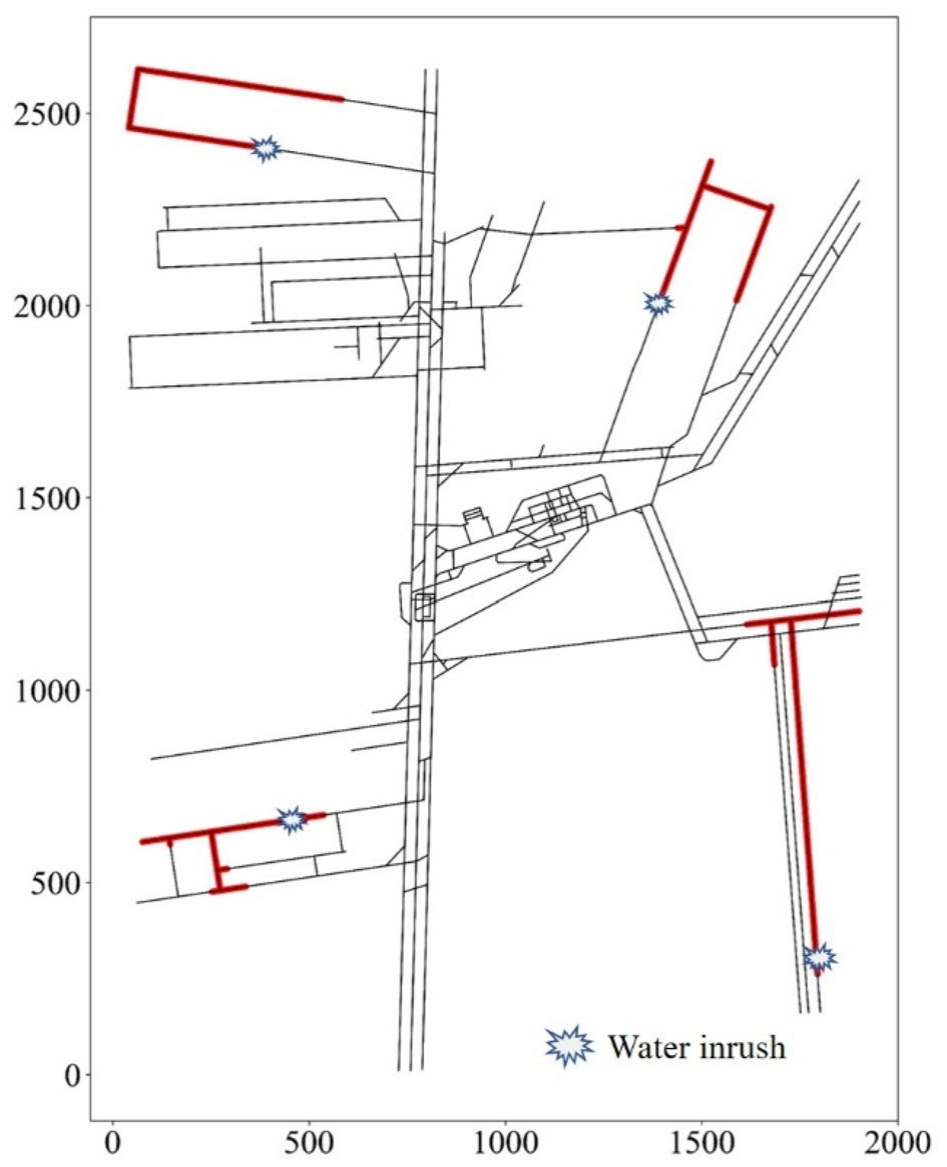
The spreading scopes of water inrush at four nodes.

**Figure 9:**
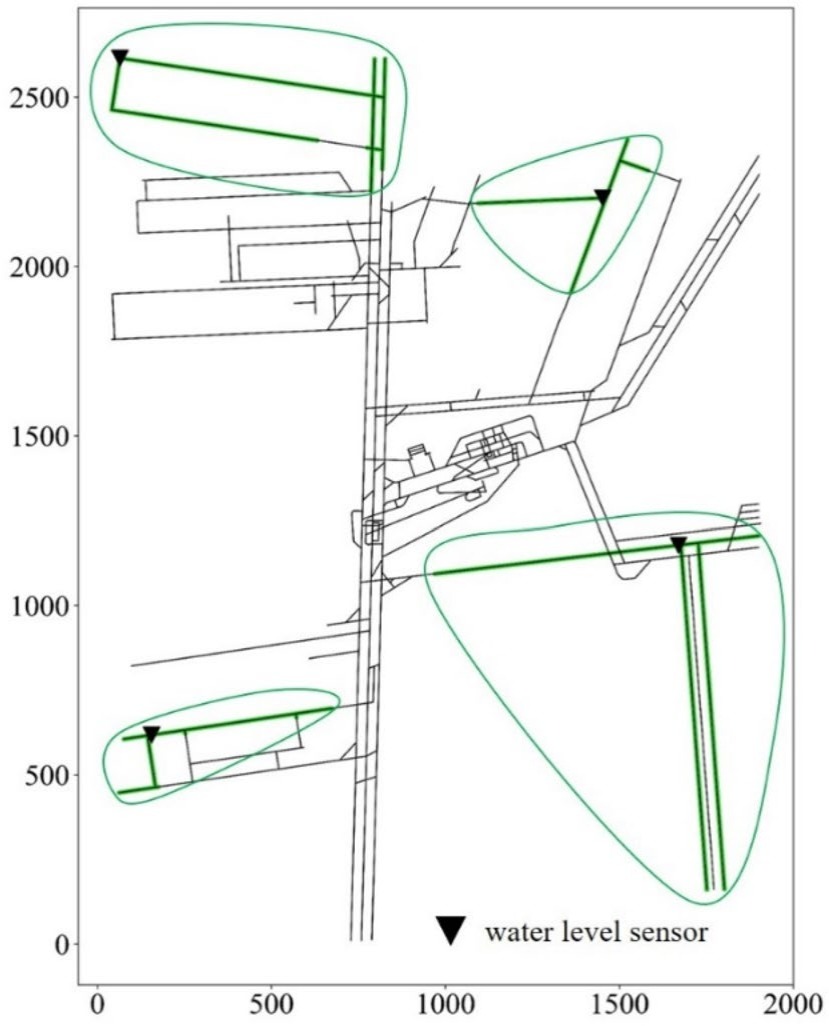
The monitoring scopes of water level sensors at four nodes.

According to the steps described in “Establishment of the set covering model” and “Solution procedures”, the set covering model of Beiyangzhuang Mine was established, and the greedy algorithm was used to solve it. The detailed steps are as follows:

1. Establishment of mine tunnel topology.
  The topology of the mine tunnels was established using the mine excavation engineering and mine tunnel traverse points (1658 points) of the Beiyangzhuang mine. The tunnel nodes were interpolated to increase the number of nodes to 8761. The set of all nodes is expressed as $N=\{0, 1, 2, 3, \dots , 8760\}$.
2. The spreading scope of water inrush.
  The value of water inrush for mining areas 1, 2, 3, and 4 were set to 800 m^3^/h, 1000 m^3^/h, 1200 m^3^/h, and 2000 m^3^/h. Similarly, the value for the remaining areas was set to 5080 m^3^/h. The water inrush process was simulated at all nodes using SWMM. The spreading scope of each node was obtained by processing the simulation data. For example, four of these results are shown in Fig. 8.
3. Determination of monitoring scope.
  The monitoring scopes of all nodes are represented as *M*, such that
  $$M=\left\{{M}_{0},{M}_{1},{M}_{2},\dots ,{M}_{j}\right\} j=\left(0, 1, 2,\dots , 8760\right).$$
  *M* can be obtained on the basis of the spreading scopes of all the nodes. Four of the monitoring scopes are shown in Fig. 9.
  Then the set covering model can be expressed as below:
  Selecting the smallest number of monitoring scopes *M*_*j*_ from *M* to “cover” the set *N*.
4. Computer solutions
  According to the procedure in “Solution procedures”, the set covering model of Beiyangzhuang mine was solved using the greedy algorithm by Python.

### Results

**Figure 10:**
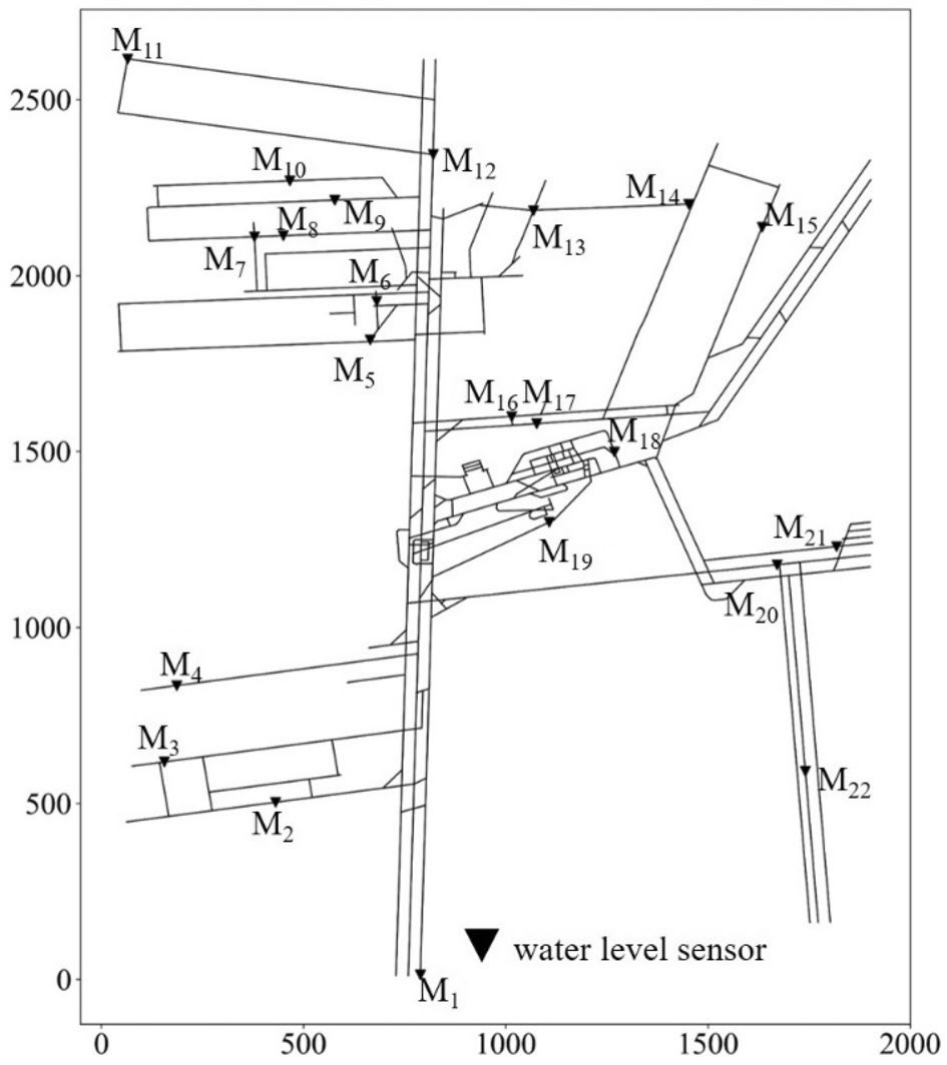
Optimal location result of water level sensors in Beiyangzhuang mine.

The number and positions of water level sensors were obtained using the optimal location method, and the result is shown in Fig. 10. There are 22 water level sensors, numbered from *M*_1_ to *M*_22_. In the results, only 6 water level sensors are located at the junctions of mine tunnels, and the remaining 16 sensors are located near local low points. The main factors affecting the selected optimal locations are the trends in the fluctuation of the tunnel height and local low-lying areas. Tunnel intersections have little influence on the optimal locations.

**Figure 11:**
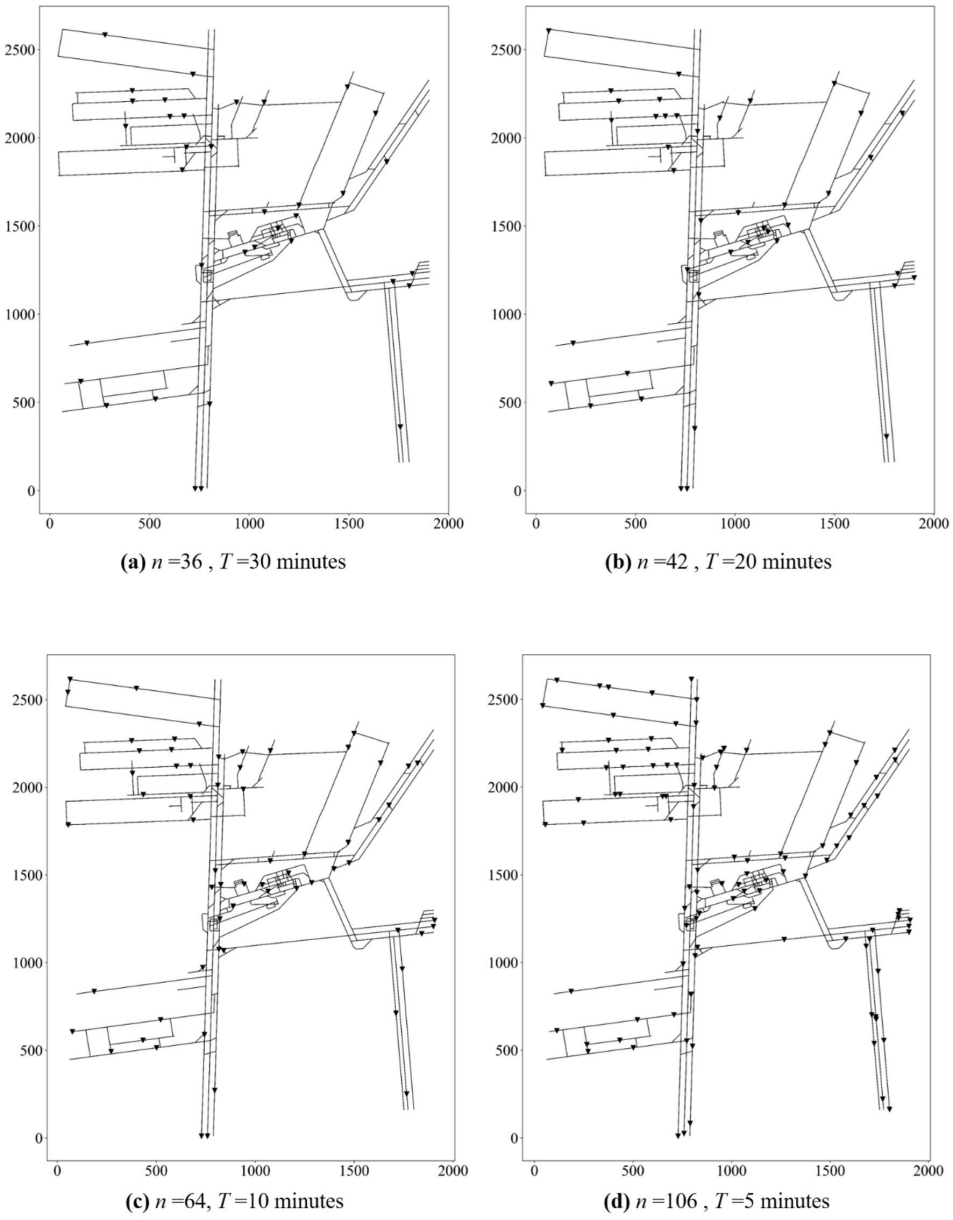
The number (*n*) and locations of water level sensors in different given time *T.*

The number (*n*) and locations of water levels sensors in different given time *T* are shown in in Fig. 11.

### Discussions

**Figure 12:**
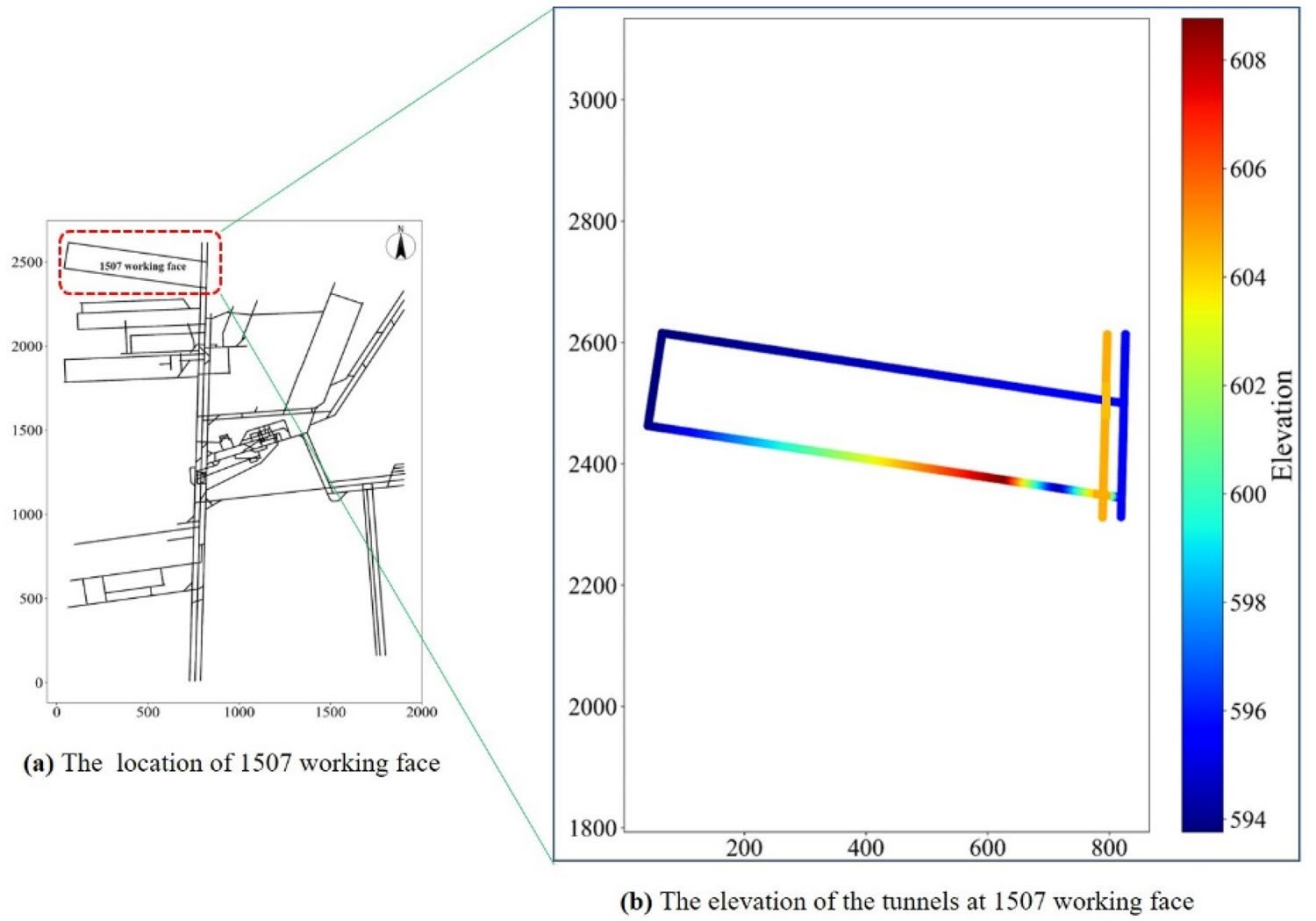
The 1507 working face in the Beiyangzhuang Mine.

In order to compare with the traditional monitoring method, the 1507 working face of Beiyangzhuang Mine was selected as the test site, and the elevation change of the tunnels’ floor was represented by gradient color, as shown in Fig. 12.

**Figure 13:**
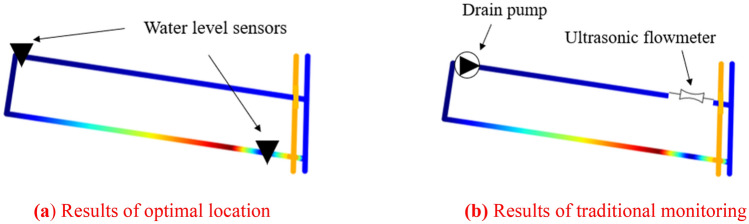
The results of location selection by the two monitoring methods.

We intercepted the location results of 1507 working face from Fig. 11b, as shown in Fig. 13a. The two water level sensors installed are directly connected to the mine industrial ring network, and the monitoring data are transmitted to the ground host in real time; The traditional monitoring scheme is mainly to install a high-power drainage pump at the low-lying part of the cut of the working face, and discharge the mine water through the drainage pipe hanging on the coal wall. An ultrasonic flowmeter is installed at the outlet of the drainage pipe to monitor the drainage volume in the drainage pipe, as shown in Fig. 13b. The ultrasonic flowmeter is directly connected to the mine industrial ring network, and the monitoring data is transmitted to the ground host in real time.

**Figure 14:**
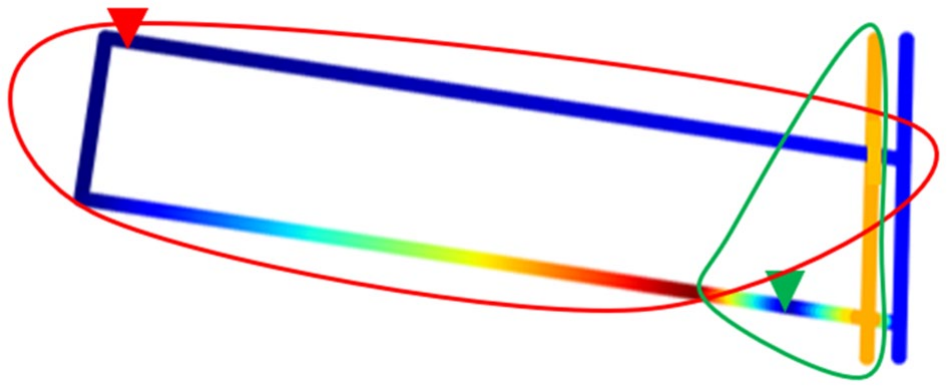
The monitoring scopes of the two water level sensors at 1507 working face.

Through the previous elaboration, it can be known that the location scheme of the water level sensor shown in Fig. 13a can monitor the water disasters of the entire working face within 20 min, and the monitoring scopes are shown in Fig. 14. The two water level sensors in this scheme are installed in the drainage ditch of the tunnels, and it monitors the depth of the water flow in the tunnels of the 1507 working face. Since the two water level sensors are directly connected to the industrial ring network, there is almost no delay in the transmission of monitoring data. The optimal location method can not only accurately calculate the monitoring scope of the water level sensor at different positions, but also inversely calculate the required monitoring response time *T* according to different location schemes.

In the traditional method of monitoring the 1507 working face shown in Fig. 13b, after a water disaster occurs, only when the drain pump is started can the accumulated water be pumped into the drainage pipeline. After the drain pump is started for a period of time, the ultrasonic flowmeter installed at the end of the drainage pipe can monitor the change of the drainage volume. This method cannot measure the monitoring scope of the ultrasonic flowmeter. Furthermore, due to the start-up time of the drain pump and the length of the drainage pipeline, the ultrasonic flowmeter cannot get the change of water disasters in the working face in time, so the monitoring response time *T* is unpredictable.

From the above discussion, it can be concluded that the optimal location method has greater advantages in timeliness and monitoring scope compared with the traditional method.

### Reliability verifications

#### Monitoring scope

**Figure 15:**
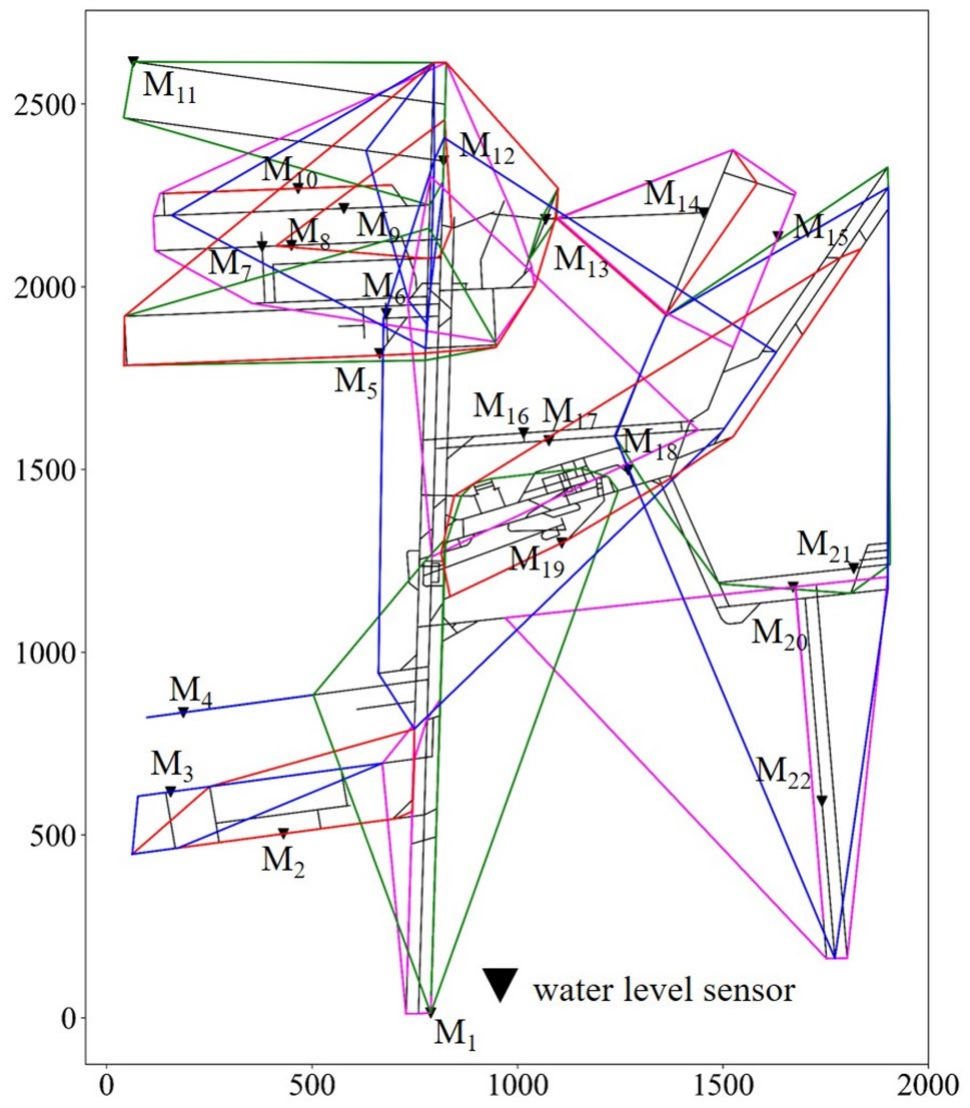
Reliability verification of the monitoring scope of the located water level sensors.

The monitoring scope of each water level sensor is displayed in Fig. 15 in the form of its smallest peripheral polygon to verify whether the water level sensors are able to monitor the entire mine. As can be seen, the combined monitoring scope of the water level sensors covers the whole mine.

#### Monitoring response time

**Figure 16:**
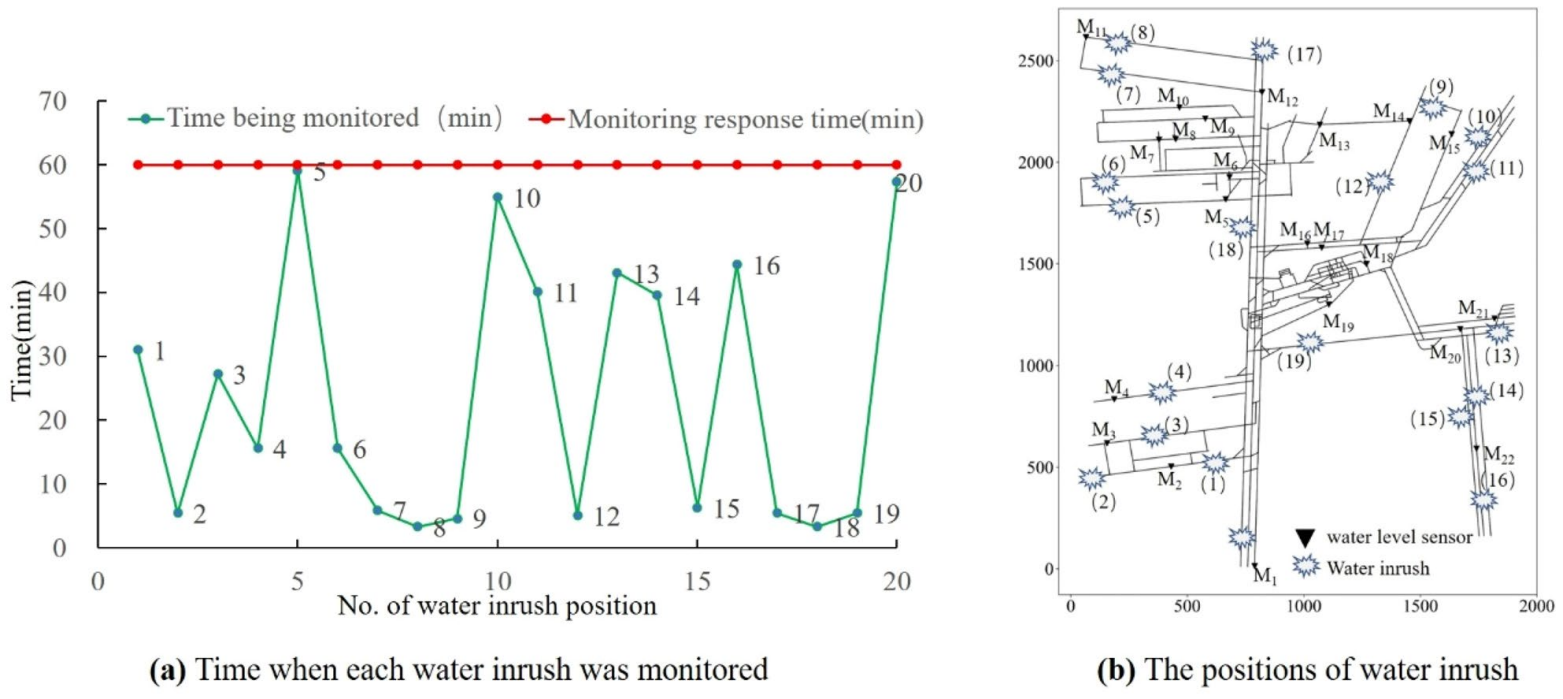
Reliability verification of the monitoring response time.

In order to test whether a water inrush accident can be monitored within the monitoring response time (60 min) based on the above location of water level sensors, the water inrush process was simulated at 20 randomly selected positions, as shown in Fig. 16b. The results show that water inrush at the above locations is monitored successfully within 60 min by the configuration of water level sensors (Fig. 16a).

## Conclusions

The development of information techniques makes it possible to monitor the occurrence and evolution of water inrush. In this paper, a new problem is introduced to arrange water level sensers to monitor water inrush that may occur in the whole range of the mine. An optimal location method is proposed to minimize the number of arranged sensors. In this process, the topology of mine tunnels is established based on the distribution of mine excavation projects and mine tunnels’ traverse points. Then, the SWMM software is used to simulate water spreading in mine tunnels.

1. A set covering model is constructed by computing the monitoring scope of the water level sensor at each location along mine tunnels. The monitoring scope is obtained from the simulated water spreading using the SWMM.
2. In the simulation of water spreading, the mine drainage capacity is used as the water inrush quantity. A greater quantity may cause water inrush disaster. Also, different drainage capacity is considered in different positions along mine tunnels.
3. Then, the greedy algorithm can be used to solve the set covering model. This method was used to optimize the location of water level sensors in the Beiyangzhuang coal mine. The results show that at least 22 water level sensors are needed to monitor water disasters in the whole mine within 60 min. And the minimum number of water level sensors required within 30, 20, 10 and 5 min is 36, 42, 64 and 106, respectively.

In the following researches, the damage and movement of water level sensors will be considered.
